# Long-Lasting Analgesia With Transdermal Fentanyl: A New Approach in Rat Neonatal Research

**DOI:** 10.3389/fphar.2022.798011

**Published:** 2022-03-17

**Authors:** Isabelle Dutriez-Casteloot, Virginie Emmanuelli, Jean-François Wiart, Annabelle Tavernier, Capucine Besengez, Laurent Storme, Véronique Houfflin-Debarge

**Affiliations:** ^1^ ULR2694 METRICS-Perinatal Environment and Health, University of Lille, Lille, France; ^2^ Department of Biology, Faculty of Sciences and Technology, University of Lille, Lille, France; ^3^ Department of Obstetrics, Jeanne de Flandre Hospital, University Hospital Center of Lille, Lille, France; ^4^ Department of Toxicology, University Hospital Center of Lille, Lille, France

**Keywords:** analgesia, animal model, transdermal fentanyl, long-lasting management, rat pup

## Abstract

**Background:** With advances in neonatal care, management of prolonged pain in newborns is a daily concern. In addition to ethical considerations, pain in early life would have long-term effects and consequences. However, its treatment remains inadequate. It was therefore important to develop an experimental model of long-lasting analgesia for neonatal research.

**Materials and Methods:** Experiments were performed in six groups of rats with transdermal fentanyl 0, 3, 12, 50, 100, or 200 μg/kg/h from second postnatal day (P2) until weaning. Assessment of analgesia was carried out at P21, with behavioral scores (ranging from 0 to 3) using a 4% formalin test. Plasma levels of fentanyl were determined by UPLC/TQD at P22. Growth rate was investigated.

**Results:** Fentanyl 100 and 200 μg/kg/h reduced scores of formalin-evoked behavioral pain. They increased time spent in pain score 0 (8 min 55 s and 6 min 34 s versus 23 s in controls) as in low pain scores 1 and 2, and decreased time in the most severe pain score 3 (19 min 56 s and 17 min 39 s versus 44 min 15 s). Fentanylemia increased in a dose-dependent manner from 50 μg/kg/h (2.36 ± 0.64 ng/ml) to 200 μg/kg/h (8.66 ± 1.80 ng/ml). Concerning growth, no difference was observed except weaker growth from P17 to P22 with 200 μg/kg/h. Clinically, we noticed no visible side effect from 3 to 100 μg/kg/h. Concomitantly, 200 μg/kg/h was responsible for ophthalmological side effects with appearance of corneal bilateral clouding in 90% pups. No difference was observed between male and female rats.

**Conclusion:** Altogether, results indicate that transdermal fentanyl 100 μg/kg/h is an efficient therapeutic for long-lasting analgesia in lactating pups. This new model provides a useful tool for protection and welfare, and future opportunity for studying long-term health consequences of sustainable neonatal analgesia.

## 1 Introduction

Today, management of both human and animal pain is of primary importance, starting from the early stages of development. Indeed, with advances in medicine, invasive procedures are increasing in Neonatal Intensive Care Units. For example, newborns requiring prolonged hospitalization are exposed daily to various kinds of stimuli eliciting acute or prolonged pain generating chronic stress (e.g., mechanical ventilation, tracheal suction, repeated heel sticks for blood samplings, and exposure to various noises) (Anand, 1998; Walker, 2014). Numerous medical procedures expose the fetus/neonate to considerable noxious experiences during application frequently followed by a local inflammation lasting for several hours or days (Debarge et al., 2010). Furthermore, life-limiting, or life-threatening conditions may lead to decision of palliative care in newborns. In that case, newborns’ comfort must be promoted and long-lasting analgesia provided.

Over the last decades, it has become evident that preterm or term newborns perceive noxious stimuli (Grunau, 2013; Walker, 2014; Fitzgerald, 2015). As a matter of fact, nociceptive pathways are already very mature at the beginning of the third trimester of pregnancy (Marchant, 2014; Victoria and Murphy, 2016). In addition, cortical responses to noxious peripheral stimulation have been reported in newborn children as young as 25 GW (Slater et al., 2010; Victoria and Murphy, 2016).

In both human and animal newborns, pain management is, thus, critical and ethically justified. In addition, there is growing evidence of long-term adverse effects and consequences of pain during the neonatal period (Grunau et al., 2006; Debarge et al., 2010; Vinall and Grunau, 2014; Walker, 2019).

Indeed, during this period of rapid growth and intensive development, the organism is particularly vulnerable to any disturbance in biological and environmental balance that impact its development. As a matter of fact, exposure to a noxious neonatal environment disrupts brain development and is responsible for chronic diseases in adulthood (Barker, 2004; Loizzo et al., 2011). Moreover, significant and lasting physiological consequences may result from painful insults in very young children related to behavioral changes (Hatfield, 2014; Walker, 2014), and also in sensitivity of neuroendocrine and immune systems to stress at maturity (Victoria and Murphy, 2016).

Together with advances in neonatal care and fundamental recognition of the right to well-being, it is important to develop an experimental model of long-lasting analgesia to provide a new tool for neonatal research. Animal models supply *in vivo* to understand physiological mechanisms in living organisms and pathological changes in most diseases. They also provide basic information for development of innovative therapeutic strategies available for both human and veterinary medicine. For example, despite differences in the type of tissue injury used in rat models of neonatal pain, findings support that evidence from animal models parallels the observations in human clinical data (Johnston and Walker, 2003; Victoria and Murphy, 2016). Moreover, provision of quality veterinary care for laboratory animals requires mastering an effective coverage of procedural pain relief. However, most of the potent analgesics have a short duration of action (1–4 h), responsible for frequent administration inducing repeated disruption of the animal. Individual responses to pain and individual variations in drug absorption do not allow bringing consistent levels of pain relief in all animals (Gaertner et al., 2008). To stop the various inconveniences, selecting a model of long-lasting and effective analgesia delivered in a homogeneous way is extremely important.

We hypothesized that transdermal fentanyl could produce optimal analgesia efficiency under chronic conditions lasting throughout lactation. Fentanyl is a high-potency synthetic opioid agonist interacting predominantly with µ-opioid receptor (Nelson and Schwaner, 2009). It is increasingly used in Neonatal and Pediatric Intensive Care Units due to its rapid onset of action, short duration of effect, effective analgesic, anesthetic properties, and relatively safe respiratory and hemodynamic profiles (Hall and Shbarou, 2009; Wolff et al., 2012; Maitra et al., 2014). Moreover, its chemical properties make fentanyl suitable for transdermal therapeutic delivery (Sathyan et al., 2005). Indeed, fentanyl patches are used in human medicine to provide pain relief for chronic pain (Hemati et al., 2015).

To answer clinical and ethical concerns in treatment of prolonged pain for newborn infants, we looked for a new experimental model of long-lasting analgesia in rats using transdermal fentanyl. For that purpose, we evaluated the ability of transdermal fentanyl to develop an analgesic model presenting therapeutic efficiency, bioavailability, and harmlessness on development of rat pups with the aim of long-lasting treatment throughout lactation. In this context, first, we determined the efficiency of fentanyl patches at different doses on pain response with the formalin test. Second, we evaluated its bioavailability by determining fentanyl plasma levels under these treatments, and at least, we studied body weight and clinical status of pups receiving transdermal fentanyl throughout lactation.

## 2 Materials and Methods

### 2.1 Ethics Statement

Animal experiments were performed at the Lille Hospital and University Department of Experimental Research according to the Amsterdam Protocol on animal protection and welfare, and directive 86/609/EEC on the protection of animals for experimental and other scientific purposes updated in the European Council, and in compliance with French law (decree 87-848, dated 19-10-1987).

The animal house (accreditation number: 59286) was placed under control of a director who is the “designated responsible person” under French law.

The experimental protocol used in the present study was examined and approved by the Ethical Committee for Experiments on Animals of Hauts-de-France region, agreement CEEA 062009, and was carried out by qualified personnel.

### 2.2 Animals

Pregnant female Wistar–Han rats (200–250 g) at 14th embryonic day (E14) were purchased from Charles River Laboratories (l’Arbresle, France). They were housed individually upon receipt in plastic cages with wood chip bedding. Animal rooms were maintained on a 12:12 h light–dark cycle. Animals were permitted free access to food (regular rat chow, A04, SAFE, Augy, France) and tap water in temperature-controlled room (22–23°C).

The dams gave birth spontaneously between E21 and E22.

### 2.3 Study Design

Transdermal patches of 12 μg/h Fentanyl^®^ (Durogesic^®^, Janssen-Cilag, Issy-les-Moulineaux) were used. Size of patches was determined, reporting body weight to expected concentration of fentanyl. Adhesive side was applied to the pup abdomen on the second postnatal day (P2). The patch was left in place for 72 h and was changed every 3 days for 20 days alternating abdominal sides for each subsequent patch application. From P14, skin was shaved prior to application.

Finally, for each application, a transparent adhesive bandage (Opsite^®^, Smith and Nephew, Le Mans) was applied over the site to maintain the patch and to avoid its oral absorption by pups or mother. Control animals received only the transparent adhesive bandage.

### 2.4 Treatments

At P2, litter size was adjusted to eight pups per dam. Newborns were then sexed (sex ratio 1:1), weighed, and identified.

Pups of the same litter were randomly assigned to one of six groups. In five experimental groups, they received transdermal patches of fentanyl at 3 (F3), 12 (F12), 50 (F50), 100 (F100), or 200 (F200) µg/kg/h every 72 h, while control pups received only an Opsite^®^.

### 2.5 Growth

Body weight was measured just before changing patches at P5, P8, P11, P14, P17, P20, and at the end of the study on P22.

### 2.6 Formalin Test (Pain Test)

For habituation, 15 min before test, rat pups were placed individually in standard transparent Plexiglas cages (30 × 20 × 20 cm) with a mirror placed behind the chamber.

Formalin (20 μl at 4%) was injected subcutaneously into the subplantar level of the left hindpaw using a 30-gauge needle.

Behavior was rated for 1 h from score calculated for each minute period. Scored behaviors were those originally described by Dubuisson and Dennis (1977). Briefly, the scores were: 0 = normal weight bearing the injected paw (i.e., foot flat on the floor with toes splayed); 1 = lameness during locomotion or resting paw lightly on the floor; 2 = elevation of injected paw so that at most, nails touch the floor; 3 = licking or biting of injected paw.

Formalin test was performed 3 weeks after birth at P21.

### 2.7 Plasma Collection

Before the sacrifice, pups were rapidly weighed and killed by decapitation. Blood samples were collected for determining fentanyl concentration at P22 in tubes pre-rinsed with 5% ethylene diamine tetra-acetic acid (EDTA) and centrifuged at 4,000 × *g* for 10 min at 4°C. Plasma samples were stored at −20°C until assayed.

### 2.8 Determination of Plasma Fentanyl Concentrations

Fentanyl plasma levels were determined by ultrahigh-performance liquid chromatography (UPLC) and triple quadrupole detector (TQD) mass spectrometry (MS) method. All the determinations were carried out at least in duplicate.

Samples were extracted in borate buffer pH 9.0 with a mix of dichloromethane/ether/hexane/isoamylalcool (300/500/200/5), and centrifuged for 5 min at 5,000 × *g*. Organic phases were injected in UPLC/TQD. UPLC separation was achieved with Acquity HSS C8 Waters column. Total cycle time was 5 min. Mass detection was performed by positive ion mode electrospray MS-MS. Fentanyl-D5 (100 ng/ml) was used as internal standard. The limit of detection for fentanyl was 0.05 ng/ml. Intra- and inter-assay coefficients of variation are estimated to be 5% and 10%.

### 2.9 Statistical Analysis

Every group consisted of 13–16 rat pups.

All results were expressed as arithmetic means ± SEM. One-way analysis of variance (ANOVA) followed by the *post-hoc* test of Tukey was performed to study data between groups.

Statistical analyses were performed using GraphPad Prism Software Version 6.0 (GraphPad Software, Inc., La Jolla, CA, USA). Significance was set at *p *< 0.05.

## 3 Results

### 3.1 Formalin Test

Assessment of analgesia effects of a long-lasting treatment with fentanyl during neonatal development was experimented 3 weeks after birth at P21 using formalin test (Dubuisson and Dennis, 1977).

**FIGURE 1:**
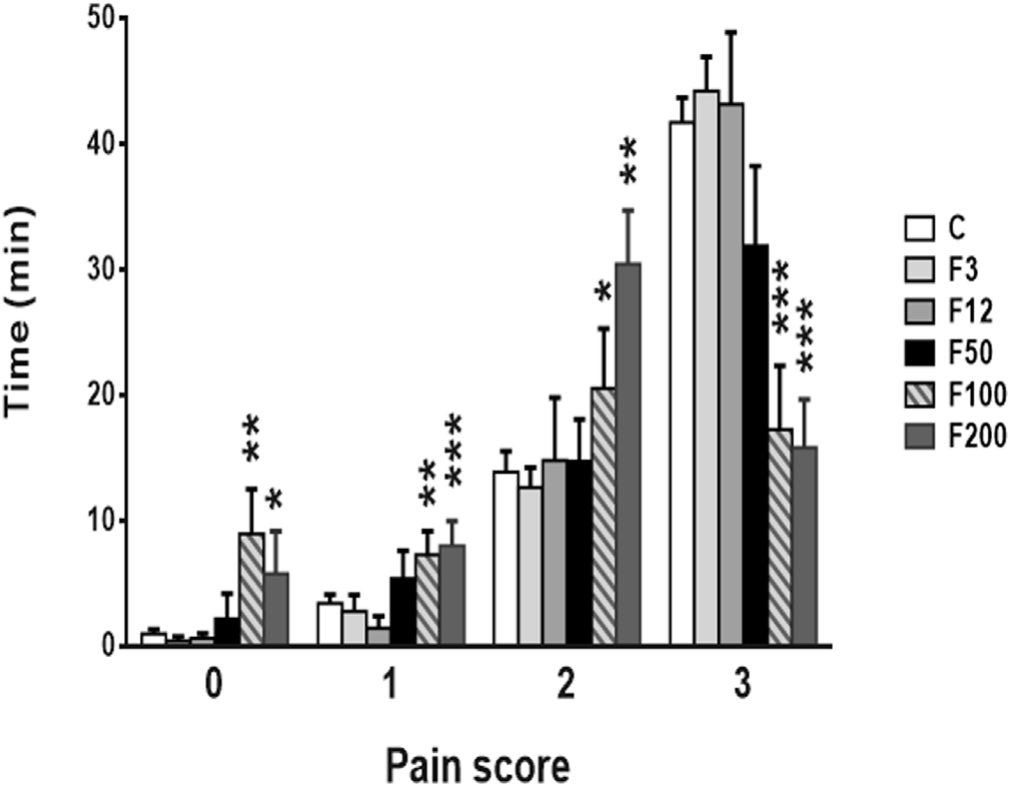
Pain assessment with formalin test 3 weeks after birth in rat pups under long-lasting analgesia. The F100 and F200 groups showed reduced scores of the formalin-evoked behavioral pain. F3: Fentanyl^®^ (F) 3 μg/kg/h (*n* = 8), F12: 12 μg/kg/h (*n* = 10), F50: 50 μg/kg/h (*n* = 10), F100: 100 μg/kg/h (*n* = 13), F200: 200 μg/kg/h (*n* = 14). C, control rats (*n* = 13). Pain scores (increasing from 0 to 3) were pooled every 5 min and expressed in means ± SEM. *p < 0.05 vs. C, **p < 0.01 vs. C, ***p < 0.0001 vs. C.

For the less severe conditions of analgesia using transdermal patches delivering fentanyl 3, 12, and 50 μg/kg/h, no difference was observed with controls. Pups spent similar times in scores of pain 0, 1, 2, and 3 (Figure 1).

Analgesia decreased behavioral responses to pain induced by injecting 4% formalin only using fentanyl patches delivering 100 and 200 μg/kg/h. Times spent in score of pain 0 were, respectively, 8 min 55 s and 6 min 34 s for fentanyl 100 and 200 μg/kg/h, respectively, and were significantly increased compared with control rats (23 s) (*p* = 0.0054, controls vs. 100 μg/kg/h; *p* = 0.0404, controls vs. 200 μg/kg/h). Similarly, the time spent in score 1 of 7 min 46 s for 100 μg/kg/h and 9 min 4 s for 200 μg/kg/h was longer than for controls (2 min 45 s) (*p* =0.0094, controls vs. 100 μg/kg/h; *p* = 0.011, controls vs. 200 μg/kg/h) as well as in score 2 with 23 min 23 s for 100 μg/kg/h and 26 min 43 s for 200 μg/kg/h fentanyl against 12 min 37 s for controls (*p* = 0.0299, controls vs. 100 μg/kg/h; *p* = 0.0032, controls vs. 200 μg/kg/h). On the contrary, rat pups exposed to 100 and 200 μg/kg/h of fentanyl patches spent a shorter time than controls in score 3 (44 min 15 s for controls and, respectively, 19 min 56 s for 100 μg/kg/h, and 17 min 39 s for 200 μg/kg/h, *p *< 0.0001) (Figure 1).

### 3.2 Plasma Fentanyl Concentrations

Fentanyl plasma levels were determined using UPLC/TQD at P22 to evaluate bioavailability of the drug in pups at weaning.

No significant differences in fentanyl concentrations were observed between male and female rat pups allowing to pool the results in each experimental condition.

**FIGURE 2:**
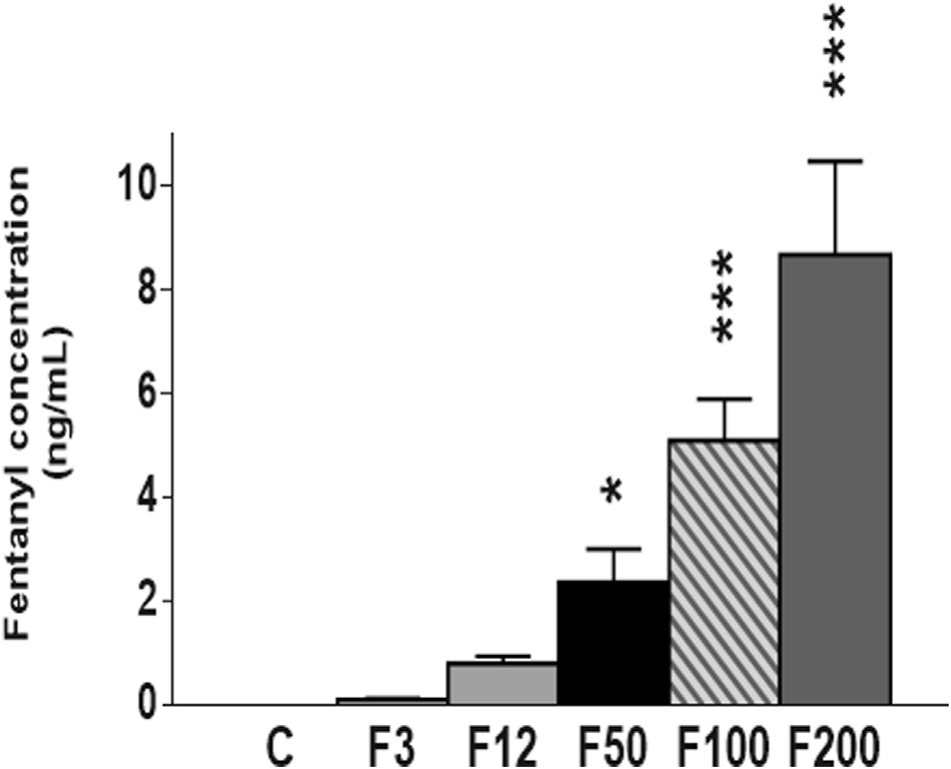
Fentanyl concentrations in plasma of rat pups were determined at postnatal day 22 (P22) by ultrahigh-performance liquid chromatography (UPLC)/triple quadrupole detector (TQD). From F50, fentanyl was detected in the plasma in a measure-dependent way (F50 < F100 < F200). F3: fentanyl^®^ (F) 3 μg/kg/h (*n* = 10), F12: 12 μg/kg/h (*n* = 12), F50: 50 μg/kg/h (*n* = 9), F100: 100 μg/kg/h (*n* = 11), F200: 200 μg/kg/h (*n* = 10). C, control rats (*n* = 13). Concentrations were expressed in ng/ml (mean ± SEM). *p < 0.05 vs C, ***p < 0.0001 vs. C.

No fentanyl was detected in control pups (Figure 2). Similarly, fentanyl plasma levels in pups that received 3–12 μg/kg/h fentanyl patches were close to zero (respectively, 0.10 ± 0.03 and 0.79 ± 0.14 ng/ml) (Figure 2).

Fentanyl amounts increased up to 2.36 ± 0.64 ng/ml for 50 μg/kg/h fentanyl patches (*p* = 0.0392, controls vs. 50 μg/kg/h). Circulating levels reached 5.09 ± 0.79 ng/ml for transdermal fentanyl 100 μg/kg/h, and 8.66 ± 1.80 ng/ml for 200 μg/kg/h (*p* < 0.0001, in both cases).

From delivered doses of transdermal fentanyl superior or equal to 100 μg/kg/h, drug plasma level was significantly different with other groups (*p* < 0.0001, 100 μg/kg/h vs. controls, 3 μg/kg/h, and 12 μg/kg/h; *p* = 0.0220, 100 vs. 50 μg/kg/h; *p* = 0.0024, 100 vs. 200 μg/kg/h; *p* < 0.0001, 200 μg/kg/h vs. controls, 3, 12, and 50 μg/kg/h) (Figure 2).

### 3.3 Postnatal Growth

Body weight was followed from P2 to P22 to understand the safety of long-lasting fentanyl treatment on development of rat pups.

No significant differences in body weight were observed between males and females at this stage of development, so we pooled the results in every experimental condition.

**FIGURE 3:**
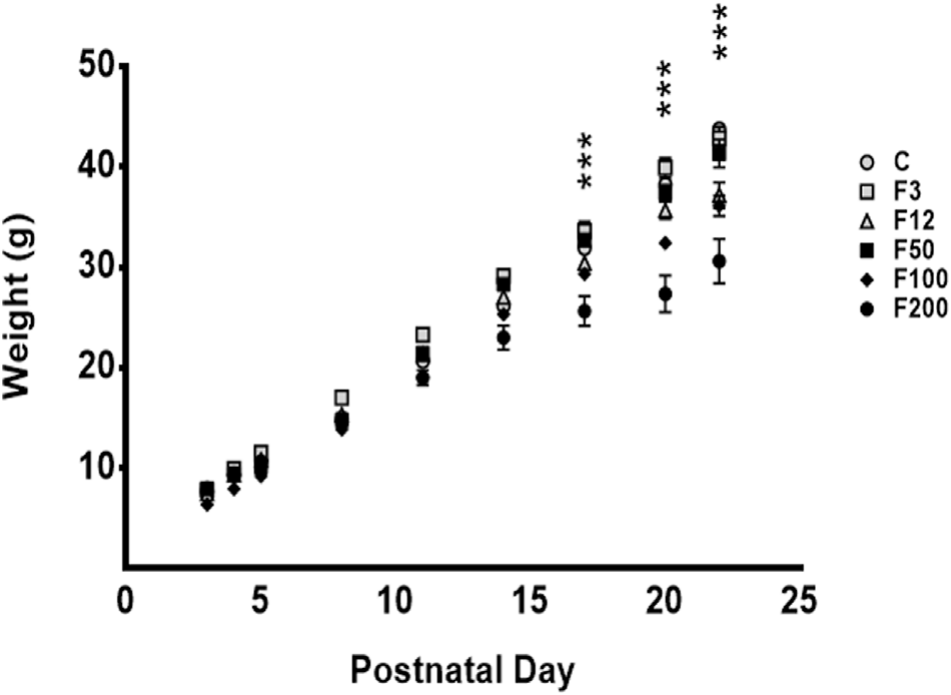
Body weight curves of rat pups exposed to fentanyl from postnatal day 2 (P2) until weaning (P22). From P17, a lower weight was observed only in the group F200. F3: fentanyl^®^ (F) 3 μg/kg/h, F12: 12 μg/kg/h, F50: 50 μg/kg/h, F100: 100 μg/kg/h, F200: 200 μg/kg/h. C, control rats. Body weights were reported in means ± SEM (g, *n* = 10–14 rats/group). ***p < 0.0001, F200 vs. C.

Until P14, the weight of the pups was comparable between the six experimental groups (*p* = 0.1824) (Figure 3).

From P17 to P22, pups that received transdermal fentanyl 200 μg/kg/h presented reduced growth compared with the other groups (Figure 3). Body weight of pups exposed to 200 μg/kg/h patches was lower than those of controls at P17, P20, as well as at P22 (*p *< 0.0001). Similarly, rat pups exposed to transdermal fentanyl 200 μg/kg/h showed significant decrease in their body weight compared with those exposed to transdermal fentanyl 3 μg/kg/h (*p* < 0.0001 at P17 and P20; *p* = 0.0002 at P22), 12 μg/kg/h (NS, *p* = 0.0718, P17; *p* = 0.0392, P20; *p* = 0.0286, P22), 50 μg/kg/h (*p* = 0.0013, P50; *p* < 0.0001, P20 and P22), and 100 μg/kg/h (*p* = 0.0351, P17; *p* = 0.0075, P20; *p* = 0.0070, P22).

During lactation, no difference was observed between the growth of controls and pups exposed to transdermal fentanyl 3, 12, 50, and 100 μg/kg/h (Figure 3).

### 3.4 Clinical Considerations

It should be notified that fentanyl did not cause any mortality in rat pups during the study.

No visible side effect related to limb or chest wall rigidity, respiratory failure, laryngospasm, local irritation, and skin reactivity to patches was observed in any of the groups treated with any dosage of transdermal fentanyl.

In addition to the weight effects mentioned above, unwanted ophthalmological effects appeared with transdermal fentanyl 200 μg/kg/h only. These adverse events consisted in the gradual development of a cornea bilateral clouding in 90% of the pups from P14 up to P22.

## 4 Discussion

Our study constitutes the very first one investigating the possibility of long-lasting analgesia in rat pups throughout lactation. In this context, we have shown that transdermal fentanyl at a 100 μg/kg/h is a good compromise between clinical effects, serum levels, and side effects.

### 4.1 What About a Long Analgesia in Early Life?

Today, pain management presents a major challenge in neonatal care. Over the last decades, numerous data demonstrated that nociceptive stimuli can be perceived *in utero* and from birth by preterm and term newborns (Walker, 2014; Victoria and Murphy, 2016). In the area of health care, this recognition has led to development of new approaches for neonates and achievement of significant results regarding infant morbidity reduction. Indeed, hospitalization provides optimal conditions for treatment of diseases or perinatal and neonatal problems (such as prematurity, low birth weight, and birth defects). However, Neonatal Intensive Care Units expose infants to many chronic noxious stimuli inducing changes in the maturation and organization of functional neural circuits with later neurodevelopmental outcomes (Fitzgerald, 2005).

Analgesia is not a topic of discussion since treating prolonged pain has become a major concern. Nevertheless, fear of long-term adverse consequences constitutes a real problem.

Adequate pain management requires a valid assessment of pain. Nociceptive signals cause a cascade of reaction leading to reflex responses and changes in physiological, hormonal, and behavioral responses. As a matter of fact, pain assessment depends on cognitive development, clinical context, and pain typology. In addition, contextual factors, such as age and health status, must also be considered. Thus, self-assessment is the gold standard for detecting the presence, type, and severity of pain. However, in nonverbal people, such as newborns, reactions in different body systems should be used as the basis for assessing pain (Eriksson and Campbell-Yeo, 2019).

Although multiple validated scoring scales are available to assess pain in a newborn (Beltramini et al., 2017), there is no standardized or universal approach to pain management (Eriksson and Campbell-Yeo, 2019). Depending on the clinical context, pain management is considered using nonpharmacological and pharmacological modalities. For mild and moderate pain, nonpharmacological methods used in pain management are safe and important interventions in Neonatal Intensive Care Units. Nonpharmacological methods include swaddling, kangaroo (skin to skin), non-nutritious suction, breastfeeding, and massage (Carter and Brunkhorst, 2017). They decrease pain scores after acute episodes of mild pain, by increasing the release of endogenous endorphins, and has a favorable impact on maternal and premature infant parasympathetic activities (Butruille et al., 2017; Ionio et al., 2021). However, these methods alone are not sufficiently effective in the treatment of prolonged and severe pain, such as palliative care. In toddlers, the pharmacological treatment of pain involves a wide spectrum of molecules with a wide range of effects to treat mild and acute to severe and prolonged pain. These molecules used in the newborn include topical anesthetics, such as EMLA (Witt et al., 2016). Depending on the method of administration (orally or rectally) acetaminophen (paracetamol), is also used in mild to moderate procedures, such as heelsticks or circumcision, or as an antipyretic (Witt et al., 2016; Carter and Brunkhorst, 2017; Vollmer, 2020). Propofol, anxiolytics/sedatives (such as barbiturate, pentobarbital), or opioids are more specially designed for the treatment of severe and prolonged pain and end-of-life pain (Witt et al., 2016; Carter and Brunkhorst, 2017; Vollmer, 2020). However, many questions remain about the risks of their long-term use. Up until recently, no alternative to long-lasting analgesia was found to treat prolonged pain being particularly present daily in newborn infants. Therefore, the immediate challenge in dealing with sustainable analgesia in early life is to reduce uncertainty about long-term consequences.

### 4.2 Why Model Long-Lasting Analgesia in Rat?

Previous data have shown that clinical management of pain in rodents is necessary. Indeed, the nociceptive pathways and pain-signaling mechanisms are highly conserved in mammals. Thus, the central pain management is completely equivalent in rodents and other research species, such as cats, dogs, and primates, and it can be managed (Linhares and Gaspardo, 2017). However, too few studies consider pain in the very young.

With advances in neonatal care and the fundamental recognition of welfare entitlement for animals, it was important to develop a new tool to model long-lasting analgesia during the neonatal period. Indeed, animal models are improving *in vivo* to understand the early physiological mechanisms in living organisms and most diseases providing fundamental information for new therapeutic strategies.

Among small laboratory animals, the rat is one of the most studied species in neonatal research because of its biological similarity to humans. Indeed, despite differences in the type of tissue damage used in rat models of neonatal pain, the results support observations parallel to those obtained in human clinical trials (Victoria and Murphy, 2016).

In veterinary clinics as well as in research centers, provisions of quality veterinary care involve careful consideration of pathology and procedural pain relief. However, most drugs have a short duration of action, individual responses to pain, and variations in drug absorption so that they do not provide consistent pain relief in rats (Gaertner et al., 2008; Gargiulo et al., 2012). Developing a model of long-lasting analgesia homogeneously administered in rat is essential for the protection and wellbeing of pups. In the future, it will allow long-term consequences of sustainable neonatal analgesia to be studied.

### 4.3 Neonatal Long-Lasting Analgesia by Using Transdermal Fentanyl

Fentanyl is a synthetic opioid agonist used as an anesthetic and analgesic in both human and veterinary medicine. In human, its analgesic potency is 50–100 times greater than that of morphine. This morphinomimetic interacts mainly with presynaptic µ-opioid receptor of the central nervous system (review in Stanley, 2014). Its physicochemical characteristics allow it to cross the blood–encephalic barrier (Terenius, 1974) and make it suitable for delivery by a transdermal therapeutic system (Sharma et al., 2016).

The use of patches facilitates constant and continuous release of fentanyl over a 72-h period, minimizing the risk of toxicity by avoiding repeated subcutaneous injections and additional painful stress (Pastore et al., 2015). In medicine, patches containing fentanyl are used to treat persistent and moderate-to-severe chronic pain around the clock (Christo and Mazloomdoost, 2008; Park et al., 2011; Hemati et al., 2015).

Fentanyl is also frequently used as an analgesic in intensive care and neonatal resuscitation due to its powerful therapeutic efficiency (Pacifici, 2015). Indeed, in pediatric resuscitation, morphinics stand out as a treatment of choice in postoperative analgesia, painful pathologies, stress prevention, and sedation of ventilated newborns (Pacifici, 2015).

Similarly, in veterinary medicine, transdermal fentanyl presents a good efficiency (Kyles et al., 1996; Lee et al., 2000; Hofmeister and Egger, 2004). Thus, fentanyl patches are currently used in the treatment of strong perioperative pains, and in the experimentation in several species (Kyles et al., 1996; Lee et al., 2000; Foley et al., 2001; Hofmeister and Egger, 2004; Christou et al., 2015; Carlson et al., 2016). Their use avoids pitfalls associated with more typical routes of administration. Moreover, analgesia sustained release ensures more stable plasma concentrations compared with peaks associated with other routes of administration (Kyles et al., 1996; Lee et al., 2000).

Transdermal fentanyl also minimizes side effects and reduces dependence on the individual compared with repeated analgesic administrations, but this galenic formulation is rarely used for managing pain in early life. In life-limiting or life-threatening conditions, neonatal palliative care can be decided with parents. In this condition, transdermal fentanyl offers long-lasting analgesia in newborns without or difficult intravenous access.

To our knowledge, no available experimental research reports a long-lasting use of transdermal fentanyl throughout lactation in rodents. Only subcutaneous administration of fentanyl was already described in neonatal period with the use of osmotic minipumps delivering the fentanyl in doses of 100 μg/kg/h to anesthetized rat pups at P6 in only 72 h (Thornton and Smith, 1997), but this procedure is not sufficient to induce prolonged analgesia during lactation. It was, therefore, interesting to study whether transdermal fentanyl can provide optimal analgesic efficiency without adverse effects under chronic conditions throughout the lactation.

### 4.4 Experimental Modeling of Long-Lasting Analgesia for Neonatal Research

#### Body Weight

As reported above, the use of 200 μg/kg/h of transdermal fentanyl was associated with normal growth to P15, followed by a disruption in growth from P17 to P22 with a significant reduction. This could probably be due to digestive and neuronal disorders, such as anorexia resulting from exposure to an overdose of the drug. In addition, it could also result in behavioral disorders, such as hyperactivity and increased locomotor activity.

Based on normal growth curves from P2 to P22, fentanyl 3–100 μg/kg/h did not appear to have a significant effect on growth. Therefore, as growth is normal under these conditions, these doses do not appear to induce immediate physiological stress related to the treatment. Thus, it appears that only fentanyl from 3 to 100 μg/kg/h is suitable for long-lasting analgesia during lactation in rat pups. However, these initial findings should be compared with the results of the formalin test.

#### Analgesic Efficiency of Transdermal Fentanyl in Early Life

The efficiency of optimal analgesic doses of fentanyl for lasting analgesia during lactation in rats was evaluated with the formalin test. Currently used in rodents as a painful stimulus, it is considered a more satisfactory model of pain than others producing phasic pain, such as hot-plate and tail-flick test, and it has more similarities with clinical pain. Introduced as a model of tonic pain, it involves moderate continuous pain generated by injured tissues (Dubuisson and Dennis, 1977). In rats, formalin generates a nociceptive behavioral biphasic response with an acute phase of short-lasting rest beginning immediately after injection and lasting 5–10 min. After a short quiescent period, continuous prolonged response begins about 15–60 min after injection. The direct chemical stimulation of pain receptors is involved in the first phase, whereas inflammation is involved in a second one (Barrot, 2012). The formalin test determines pain in central and peripheral components (Sajedianfard et al., 2005). The first phase is produced by peripheral activation of C-fiber afferent nociceptors with formalin. The second one is due to central sensitization of spinal horn neurons resulting in initial barrage of input from C-fiber nociceptive afferents during the early phase (Gong et al., 2014).

In our study, all rat pups exposed to a 20-day lasting analgesia by transdermal fentanyl present biphasic response depending on drug dosage. If lower doses (3–50 μg/kg/h) are ineffective for nociceptive acute stimulus, fentanyl 100 and 200 μg/kg/h significantly reduces formalin-evoked pain behavior scores. Indeed, compared with control, fentanyl 100 and 200 μg/kg/h decrease pain induced by formalin increasing scores (0–2) from normal to low pain behavior and decreasing score 3 related to licking, biting, and flinching of the affected hindpaw. Thus, the central effect of transdermal fentanyl 100 and 200 μg/kg/h induces considerable analgesia in rat pups. Therefore, these two dosages may be considered efficient in performing long-lasting analgesia throughout rat lactation in neonatal research.

#### Side Effects of Sustained 20-Day Analgesia During Lactation

As with all opioids, the use of fentanyl is related to a risk of clinically significant respiratory depression. Moreover, the most common side effects in humans also include metabolism, nutrition, gastrointestinal, cardiac, psychiatric, nervous, and general disorders.

In addition to the previously reported effects on weight, adverse ophthalmological effects occurred with 200 μg/kg/h of fentanyl initially occurring as a bilateral corneal clouding in 90% of pups. To our knowledge, such an ophthalmic complication has never been described and is quite difficult to explain. Extremely rare, suspected side effects previously described include ocular disorders, such as miosis, blurred vision probably due to central disorders, or increased intracranial pressure, amblyopia, dry mouth, difficulty urinating, and constipation. Thus, it is possible that in early life, this overdose of fentanyl alters the fluid and electrolyte balance leading to the development of a bilateral corneal clouding. It is also known that in rats, P1 to P4 and P14 to P22 is a critical stage for corneal development, respectively, corresponding to cellular proliferation of the stroma and endothelium of the cornea and central epithelial cornea (Van Cruchten et al., 2017). Based on our observations, we think that bilateral corneal clouding occurred shortly after the eyelids were opened. Thus, this deleterious effect of 200 μg/kg/h fentanyl could be correlated with abnormal corneal development during cellular proliferation of the central epithelium and when endothelial cells continued to flatten, and the Descemet membrane thickened (Vrolyk et al., 2018). Indeed, this latter side effect in rats is very different from that observed in humans in adulthood.

At the same time, up to a dose of 100 μg/kg/h of fentanyl, none of the pups treated from P2 to P22 died. Their growth was like that of the controls, and no visible side effect was observed on cardiorespiratory, digestive, or neurologic systems. Therefore, despite the reservations mentioned above (Sajedianfard et al., 2005), transdermal patches are well suited for experimentation in rats due to easy handling in pups from P2 to P22. Moreover, applying patches induced no skin irritation or reactivity. Furthermore, we have shown that fentanyl can be administered continuously through rat skin to enter the systemic circulation of pups reaching therapeutic concentration.

#### Therapeutic Fentanyl Levels

Assessing appropriate analgesic therapy throughout lactation, doses of fentanyl were selected from those recommended by others using different routes of administration and not only using the transdermal formulation (Thornton and Smith, 1997; Mencía et al., 2007). Our data in rat pups show that plasma levels of fentanyl increase with doses of fentanyl in the patches (3–200 μg/kg/h) and, therefore, with all doses delivered, even the lowest. The most significant effects of fentanyl release and skin penetration were observed primarily for 50, 100, and 200 μg/kg/h of Durogesic^®^ with plasma rates of fentanyl ranging from 2.36 to 8.66 ng/ml, respectively. No sex-related differences were found, probably due to the underdevelopment of subcutaneous fat in rat pups (Ohtsuka et al., 2007).

In humans at adulthood, serum levels of fentanyl causing minimal analgesic effect fluctuate between 0.3 and 1.5 ng/ml in opioid-naive patients (European Monitoring Center for Drugs and Drug Addiction, 2021). In addition, the recommended serum concentration is 1–2 ng/ml for analgesia and 10–20 ng/ml for anesthesia (European Monitoring Center for Drugs and Drug Addiction, 2021). Moreover, in humans, blood concentrations of approximately 7 ng/ml or greater were associated with fentanyl-related deaths where poly-substance use was involved (Carpenter et al., 2020). Although, cases have been reported after therapeutic use, many deaths have occurred due to misuse of drugs (injected, smoked, snorted, or taken orally).

In our experiments, plasma levels of fentanyl 5.09–8.66 ng/ml induce optimal long-lasting and continuous analgesia throughout the lactation in rat pups as shown by the formalin test. However, under these conditions, plasma levels of fentanyl were 5- to 10-fold higher than those responsible for optimal analgesic efficacy of the drug and more closely correlated with the anesthetic concentration of fentanyl in adult humans (European Monitoring Center for Drugs and Drug Addiction, 2021). In fact, the concentration required in humans for analgesia is commonly extrapolated to animal species and particularly to pigs (Wilkinson et al., 2001). For example, Foley et al. reported that rabbits treated with transdermal fentanyl had concentrations within the range considered analgesic in humans with a mean value of 0.83 ng/ml obtained between 12 and 72 h (Foley et al., 2001). This difference in efficacy of the optimal plasma levels we observe may be due to dose effect, differences in skin characteristics between rat pups and human adults, and/or more likely metabolic differences between immature and mature organisms, lower efficiency of the enzymatic system, and/or to a shorter half-life of fentanyl in rat pups than in humans. However, this would not explain the differences in dose effects since at these concentrations, we found a dose-dependent effective response on the formalin test. Thus, no concrete explanation has been provided at this time but the difference in optimal concentration may be related to the species, stage of development, or size of rat pups. Existence of a transcutaneous crossing allows the possibility of modeling long-lasting analgesia using transdermal fentanyl in rat pups throughout lactation. In addition, unlike studies on other species (e.g., rabbits), we did not encounter problems with abundant hairs, as they were underdeveloped in rat pups (Foley et al., 2001). Furthermore, specific skin characteristics play a role in the percutaneous adsorption of the drug and its rate of delivery. Thus, despite previous observations, fentanyl patches are well suited for pain management in newborn rats due to an easy handling of the pups from P2 to P22.

Finally, by looking at effective serum levels from the perspective of analgesia in human clinics, we observed that in rat pups, transdermal fentanyl 50 μg/kg/h is associated with a plasma level of 2.36 ng/ml close to the effective analgesic concentration in humans. However, this one has no significant therapeutic effect in the formalin test. It is possible that fentanyl at this concentration may have an effective analgesic effect on chronic or repeated mild pain not modeled in the formalin test.

Thus, sustained analgesia during lactation may be relieved by providing a dose of transdermal fentanyl between 50 and 200 g/kg/h depending on the stimulus. Based on our results, fentanyl 100 μg/kg/h is a good compromise with no significant side effect and may be suitable for long-lasting analgesia during lactation in rat pups. Our data constitute the very first study exploring the possibility of long-lasting analgesia in rats throughout lactation. In the future, further studies can examine the long-term consequences of chronic neonatal exposure to opioids to examine the potential effects of iatrogenic tolerance and dependence in human infants.

#### Limits of the Study

Our study concerns the development of a translational experimental model at the interface of human and veterinary clinics. The main interest for us at this stage was not to characterize the effects of fentanyl in the rat but to develop a model to answer the questions posed by the long-term effects of sustained analgesia with fentanyl during lactation.

In this context, we only considered therapeutic efficacy, bioavailability, and clinical and behavioral criteria to support the validity of our model. Indeed, we relied on these aspects because clinically, numerous scales have been validated to specifically assess acute or prolonged pain (Beltramini et al., 2017). Thus, for children under 6 years of age, behavioral pain scales are needed to assess pain. Nevertheless, facial expressions, body movements, and physiological changes are not unique to pain. Depending on physiological and neurophysiological responses, biomarkers can be used as indicators of pain and comfort in infants (De Jonckheere and Storme, 2019; Eriksson and Campbell-Yeo, 2019). In fact, pain assessment may consider biomarkers alone or in combination with behavioral response. Thus, newborn pain can be assessed based on the assessment of behavioral pain and heart rate variability (Rakza et al., 2018). In addition, parasympathetic neonatal assessment (NIPE) may be considered a predictor of hemodynamic response in children under 2 years of age (Faye et al., 2010; De Jonckheere et al., 2011; De Jonckheere and Storme, 2019; Zhang et al., 2019). Here, we extrapolated the clinical results with parasympathetic activity for reasons previously discussed (weight, respiration… ). Finally, it should be noted that in our procedure, we observed a combination of pharmacological and nonpharmacological approaches since the effect of fentanyl is inseparable from that of maternal behavior.

## 5 Conclusion

Our work has been to develop an analgesic model with therapeutic efficiency, bioavailability, and harmlessness on growing rats for long-lasting treatment. Overall, our data showed that transdermal fentanyl at 100 μg/kg/h can be selected based on its therapeutic proprieties. Our results are the first ever study investigating the possibility of long-lasting analgesia in rats throughout the period of lactation. In the future, research carrying on neonatal pain and its consequences could be based on this original work.

## Data Availability

The raw data supporting the conclusions of this article will be made available by the authors, without undue reservation.
